# Hyperoside exerts osteoprotective effect on dexamethasone-induced osteoblasts by targeting NADPH Oxidase 4 (NOX4) to inhibit the reactive oxygen species (ROS) accumulation and activate c-Jun N-terminal kinase (JNK) pathway

**DOI:** 10.1080/21655979.2022.2054499

**Published:** 2022-03-24

**Authors:** Siqi Fan, Haida Pan, Jiaxing Huang, Zhiqiang Lei, Jinfu Liu

**Affiliations:** a Department of Orthopedics and Traumatology, The First Affiliated Hospital of Guangxi University of Chinese Medicine, Nanning, China; b Research Student Academy, Guangxi University of Traditional Chinese Medicine, Nanning, China; c Yulin Orthopedics Hospital of Chinese and Western Medicine, Yulin, China; d Emergency Department, The First Affiliated Hospital of Guangxi University of Chinese Medicine, Nanning, China

**Keywords:** Hyperoside, NADPH oxidase 4, osteoblast, osteonecrosis of the femoral head

## Abstract

Hyperoside (Hyp) is a flavonoid active compound deriving from Chinese herbal medicines. Increasing studies have implicated that Hyp may serve as a predominant promoting factor in osteoblast differentiation. This paper investigates whether Hyp could relieve glucocorticoid-induced osteonecrosis of the femoral head (GONFH) via promoting osteoblast survival and differentiation as well as to uncover its potential mechanism. GONFH cell model was induced by treating MC3T3-E1 cells with dexamethasone (DEX). The viability, apoptosis, and osteogenic differentiation of DEX-induced cells with the presence or absence of Hyp were assessed by CCK-8, Tunel, ALP assay, and ARS staining, respectively. The NADPH Oxidase 4 (NOX4) overexpression was performed by transfection with overexpression vector. Besides, western blot was used to determine the levels of apoptosis-, osteogenic differentiation-, and c-Jun N-terminal kinase (JNK) signaling-related proteins. It was noticed that Hyp caused no significant effects on the viability of MC3T3-E1 cells without any treatment but significantly enhanced the viability of DEX-induced cells. Besides, Hyp inhibited the apoptosis in DEX-induced cells but enhanced ALP activity and calcium nodule formation. Additionally, Hyp declined NOX4 expression in DEX-induced cells. However, NOX4 overexpression partially reversed the impacts of Hyp on DEX-exposed MC3T3-E1 cells. Finally, Hyp suppressed the activation of ROS/JNK pathway in DEX-induced cells, which was then counteracted by NOX4 overexpression. In conclusion, Hyp could promote the survival and differentiation of DEX-induced osteoblasts by targeting NOX4 to inhibit the ROS/JNK pathway. These results provide evidence for the application of Hyp in treating GONFH.

## Graphical abstract

**Figure:**
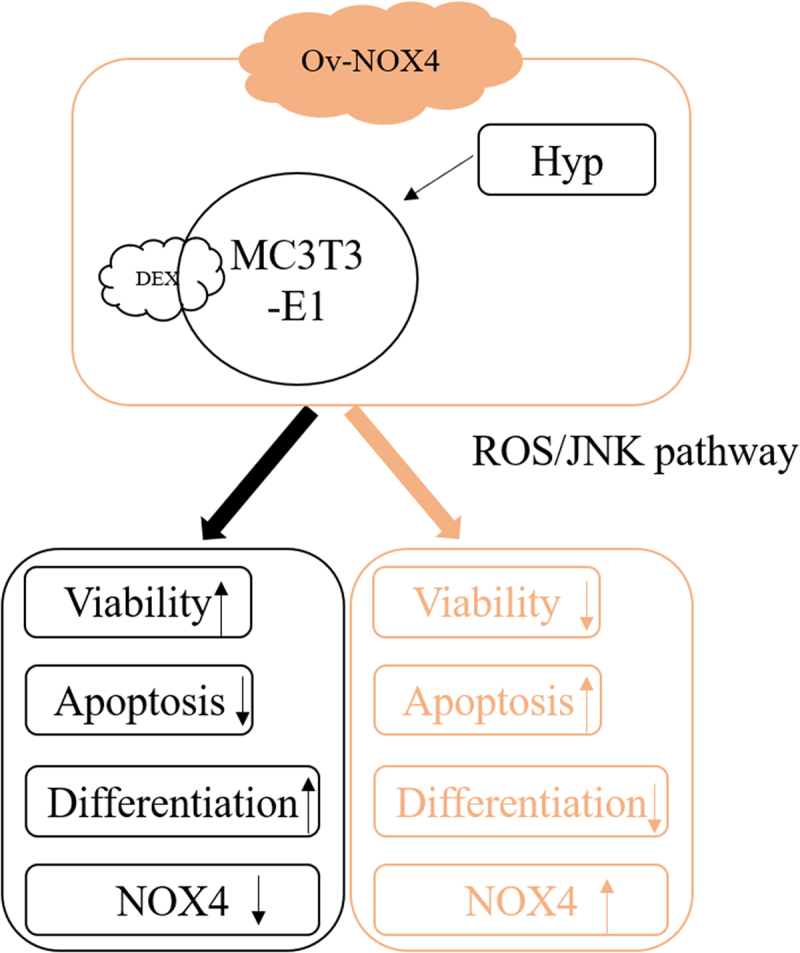

## Background

Osteonecrosis of the femoral head (ONFH) is characterized by the aggressive necrosis of bone cells and the bone marrow, with an estimated 20,000 to 30,000 new cases diagnosed each year [1]. Glucocorticoid-induced ONFH (GONFH) is a major metabolic disorder caused by long-term use of hormone drugs. GONFH can reduce vascular supply to femoral head and bone cells, ultimately leading to the dysfunction of the femoral head and hip joint [2]. Despite the fact that the underlying molecular mechanisms of GONFH remain unclear, the influences of glucocorticoid on aberrant proliferation, differentiation, and apoptosis of osteoblast and osteoclast, as well as dysfunction of lipid metabolism have been accepted [3]. Current treatment for GONFH mainly focuses on preventing against irreversible complications, including femoral head collapse and hip osteoarthritis. At the moment, the therapeutic efficacy is limited, in view of this, great efforts need to be made to develop novel effective agents [4].

Hyperoside (Hyp) is a flavonoid active compound widely found in many Chinese herbal medicines and has been documented to yield tremendous activities on cancers, fibrosis, inflammation, etc [^5–7^]. A previous study reported that Hyp could stimulate the osteogenic capacity of osteosarcoma cells and suppress cell viability [8]. The beneficial effects of Hyp on bone metabolism in vivo was found by Yiqing et al. [9]. Recently, Hyp was reported to ameliorate periodontitis in rats by promoting osteogenic differentiation of bone mesenchymal stem cells [10]. These studies indicated that Hyp may play an important role in promoting osteoblasts differentiation. Intriguingly, several natural flavonoids, such as luteolin [11], naringin [12], and pinocembrin [13], were reported to show protective effects against GONFH. Nevertheless, whether Hyp participates in GONFH has not been studied.

SwissTargetPrediction online database predicted that Hyp could target NADPH Oxidase 4 (NOX4), which is a critical source of reactive oxygen species (ROS) production. NOX4 has been recently showed to be associated with ONFH via ROS/JNK signaling [14,15]. Therefore, this study was carried out to investigate whether Hyp could regulate ROS/JNK signaling to alleviate GONFH via targeting NOX4.

This paper was aimed to investigate the treatment of Hyp targeting NOX4 on dexamethasone-induced osteoblasts through ROS/JNK signaling. We speculated that Hyp protected dexamethasone-induced osteoblasts by targeting NOX4 through inhibiting to inhibit the ROS/JNK signaling pathway. Dexamethasone-induced MC3T3-E1 cells were treated with the different concentrations of Hyp and biologic behaviors of MC3T3-E1 cells were detected. Ov-NOX4 transfected MC3T3-E1 cells were pre-treated with indicated concentration of Hyp and then treated with DEX to see whether NOX4 upregulation could affect the effect of Hyp on the biologic behaviors of dexamethasone-induced MC3T3-E1 cells.

## Materials and methods

Cell culture and treatment

The culture medium for MC3T3-E1 cells (ATCC) was MEM medium (Gibco) decorating with 10% fetal bovine serum (FBS) and 1% antibiotics. The cultivation condition was 37°C with 5% CO_2_. To construct GONFH cell model, MC3T3-E1 cells were exposed to 1 μM dexamethasone (DEX; ≥97%; Sigma-Aldrich) for 24 h. For Hyp (≥98%; Chengdu Gelipu Biological Technology Co., Ltd.) treatment, cells were exposed to 10, 25, and 50 μM of Hyp for 24 h. For DEX + Hyp treatment, cells were pre-treated with indicated concentration of Hyp for 2 h and then treated with 1 μM DEX for 24 h.

Cell transfection

To up-regulate NOX4 (Ov-NOX4), full length of human NOX4 cDNA was inserted into pcDNA3.1 vector (ThermoFisher) and blank vector pcDNA3.1 was regarded as negative control (Ov-NC). The confluence of cells reached 75%, and the culture medium was removed and replaced with a pure culture medium without serum and antibiotics. Transfection of plasmids was executed using Lipofectamine 2000 (Invitrogen) in the light of the manufacturer’s protocol. The cells were incubated for 6 h and the culture medium was replaced with normal culture medium (with serum and antibiotics) to continue culture. After transfection for 24 h, cells were harvested for the following assays.

Cell viability assay

Cell viability was estimated by CCK-8 assay. After MC3T3-E1 cells (5000 cells/well) were inoculated in a 96 well plate and maintained overnight at 37°C, 10 μL of CCK-8 solution (Promega, Madison, WI) was dropped and the cells were incubated for another 2 h at 37°C. The OD value was examined at 450 nm by a microplate reader (Beckman, CA, USA).

TdT‑Mediated dUTP Nick‑End Labeling (Tunel)

Tunel assay was carried out with the adoption of the In-Situ Cell Death Detection Kit (Roche, Basel, Switzerland). In a word, after the fixation in 4% paraformaldehyde at room temperature for 30 min, MC3T3-E1 cells were permeated by 1% Triton X-100/10 mM PBS for 5 min. Cells were double-stained by 50 μL of TUNEL detection solution at 37°C for 1 h. DAPI was used to stain the nucleus. Finally, images were captured under a fluorescence microscope (Olympus, USA).

Western blot

Protein lysates were isolated from MC3T3-E1 cells using RIPA buffer (Beyotime) and then subjected to 12% SDS-PAGE with equal amount per lane, after which it was transferred onto PVDF membranes impeded with 5% skim milk. Then, membranes were cultivated overnight at 4°C with primary antibodies (Abcam) including anti-Bcl-2 (1:5000), anti-Bax (1:5000), anti-cleaved-caspase 3 (1:2000), anti-caspase 3 (1:2000), anti-Runt-related transcription factor 2 (Runx2; 1:1000), anti-Osterix (Osx; 1:1000), anti-Collagen I (1:1000), anti-receptor activator nuclear factor κB ligand (RANKL; 1:1000), anti-NOX4 (1:2000), anti-JNK (1:5000), anti-phosphorylated (p)-JNK (1:5000), anti-c-Jun (1:5000), anti-p-c-Jun (1:5000) and anti-GAPDH (1:5000), followed by cultivation with HRP-conjugated anti-rabbit secondary antibody (Abcam; 1:5000). GAPDH served as the internal control. The proteins were detected by ImageQuant LAS 4000 (GE, USA) and quantitative analysis of the western blot was conducted with the aid of ImageJ software.

Alkaline phosphatase (ALP) activity

The activity of ALP was measured using ALP Assay Kit (Abcam) according to the protocol of manufacture. Briefly, after indicated treatment, MC3T3-E1 cells were incubated with 1 mg/mL p-nitrophenyl phosphate (pNPP, substrate) or substrate-free conditioned medium (used as standards) for about 1 h. Then, the absorbance at 405 nm was analyzed by a microplate reader after the supplementation of the stop solution.

Alizarin red S (ARS) staining

ARS staining was performed to measure osteogenic differentiation. Briefly, MC3T3-E1 cells were fixed with 4% paraformaldehyde for 15 min, and then stained with 0.2% ARS solution (Solarbio) for 20 min. After the rinse with ddH_2_O_2_, cells were observed under a microscope (Olympus, Japan).

RT-qPCR

The total RNA isolated from MC3T3-E1 cells using TRIZOL buffer (Thermo Fisher Scientific) was converted into cDNA through reverse transcription using PrimeScript™ RT reagent Kit with gDNA Eraser (Takara, Japan). Then, qPCR was executed using TB GreenTM Premix Ex TaqTM II (Takara) on a StepOnePlus Real-Time PCR system (Applied Biosystems, USA). With the employment of 2^−ΔΔCT^ method, relative mRNA levels were calculated after normalization to GAPDH. Primers (forward and reverse) are listed as follow: NOX4, 5’-CTCAGCGGAATCAATCAGCTGTG-3’, and 5’-AGAGGAACACGACAATCAGCCTTAG-3’. GAPDH, 5’-AATGGGCAGCCGTTAGGAAA-3’, and 5’-GCGCCCAATACGACCAAATC-3’.

ROS level measurement

ROS level in MC3T3-E1 cells was measured by DCFDA/H2DCFDA-Cellular ROS Assay Kit (Abcam). Briefly, cells were plated into 96-well plates (2 × 10^4^ cells/well) and incubated overnight for adherence. After the indicated treatment, 100 μL DCFDA was added to each well and incubated at 37°C for 45 min. The Ex/Em = 485/535 nm was finally measured by a fluorescence plate reader.

Statistical analysis

All experiments were conducted in triplicate. For multiple groups’ comparison, one-way ANOVA with Bonferroni’s posttest was used on GraphPad software. Results were displayed as mean ± SD. P < 0.05 was deemed to be significant.

## Results

Hyp inhibits DEX-induced osteoblast apoptosis in MC3T3-E1 cells

**Figure 1.**
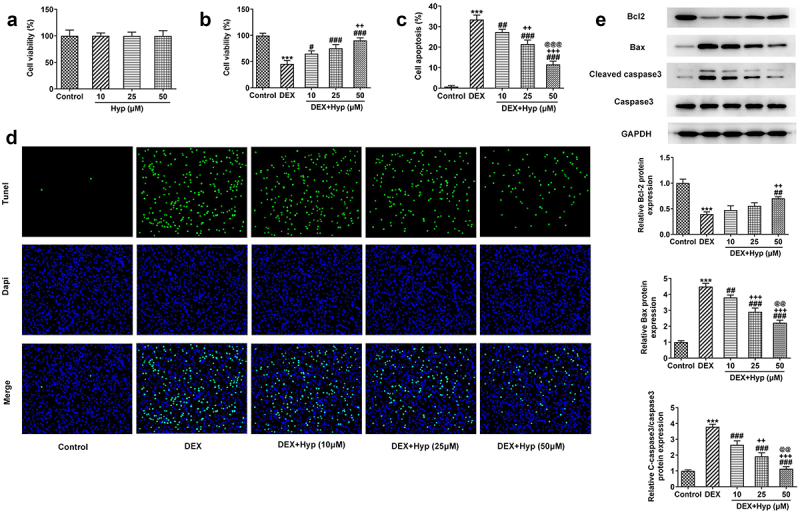
Hyp improves cell viability and inhibits apoptosis in DEX-induced MC3T3-E1 osteoblasts.A, MC3T3-E1 cells were administrated with different concentrations of Hyp for 24 h or not, and then cell viability was appraised by CCK-8. B-E, MC3T3-E1 cells were treated with control medium or 1 μM DEX, or DEX plus different concentrations of Hyp for 24 h, and then (B) cell viability was assessed by CCK-8; (C-D) cell apoptosis was observed by Tunel (x200); (E) western blot tested the protein levels of Bcl2, Bax and cleaved caspase 3/caspase 3. ***P < 0.001 vs control; ^#^P < 0.05, ^##^P < 0.01 and ^###^P < 0.001 vs DEX. ^++^P < 0.01 and ^+++^P < 0.001 vs DEX+Hyp (10 μM). ^@@^P < 0.01 and ^@@@^P < 0.001 vs DEX+Hyp (25 μM).

Firstly, to explore the impacts of Hyp on the viability of MC3T3-E1 osteoblasts, different concentrations of Hyp (10, 25, and 50 μM) were used to treat MC3T3-E1 cells. As shown in Figure 1(a), Hyp had no significant effects on the viability of MC3T3-E1 cells, indicating the safety of Hyp at these concentrations. Results in Figure 1(b) revealed that the cell viability was remarkably reduced upon DEX treatment, but Hyp significantly recovered the viability of DEX-treated cells, suggesting that Hyp protected osteoblasts from DEX-induced viability damage. On the contrary, results presented in Figure 1(c,d) illustrated that DEX caused a marked increase in cell apoptosis, which was then reduced by Hyp administration. In addition, results in Figure 1(e) showed that BCl2 expression was decreased, while Bax and cleaved caspase3/caspase3 protein expressions were increased after DEX treatment. However, Hyp treatment exhibited opposite effects on these proteins. The above data suggested that Hyp could inhibit DEX-induced cell viability impairment and cell apoptosis in osteoblasts.

Hyp promotes DEX-suppressed differentiation in MC3T3-E1 osteoblasts in a concentration-dependent manner.

**Figure 2.**
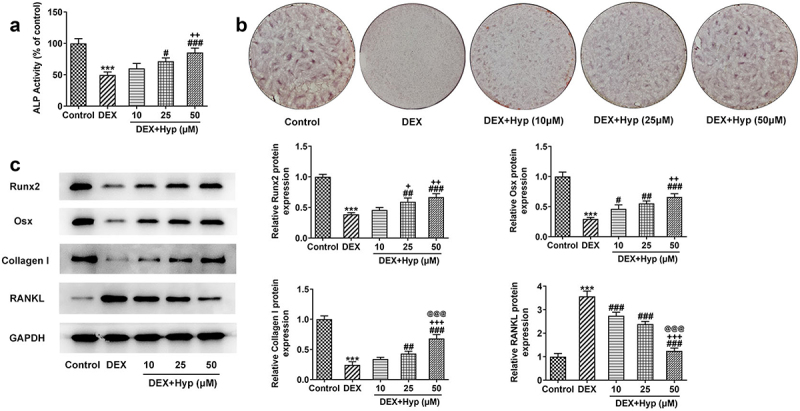
Hyp promotes osteogenic differentiation in DEX-induced MC3T3-E1 osteoblasts.A, ALP assay measured ALP activity. B, ARS staining examined osteogenic differentiation. C, western blot tested the protein levels of Runx2, Osx, Collagen I and RANKL. ***P < 0.001 vs control; ^#^P < 0.05, ^##^P < 0.01 and ^###^P < 0.001 vs DEX. ^+^P < 0.05, ^++^P < 0.01 and ^+++^P < 0.001 vs DEX+Hyp (10 μM). ^@@@^P < 0.001 vs DEX+Hyp (25 μM).

Subsequently, osteogenic differentiation of MC3T3-E1 cells was explored. Figure 2(a) showed that DEX greatly reduced the ALP activity of MC3T3-E1 cells when compared with that in cells without any treatment, while this outcome was then remarkably enhanced by Hyp co-treatment. In addition, DEX induction decreased the number of calcium nodule in MC3T3-E1 cells, which was contrarily increased upon Hyp when compared with the DEX group (Figure 2(b)). It was also found that DEX resulted in the downregulated expressions of Runx2, Osx, and Collagen I, but upregulated RANKL expression (Figure 2(c)), indicating the inhibition of osteogenic differentiation. Oppositely, cells that co-treated with DEX plus Hyp exhibited increased expressions of Runx2, Osx, and Collagen I, but decreased expression of RANKL (Figure 2(c)). These results elucidated that DEX suppressed the osteogenic ability of MC3T3-E1 osteoblasts, and this effect was restored by Hyp.

NOX4 overexpression reverses the impacts of Hyp on MC3T3-E1 osteoblasts survival and differentiation.

**Figure 3.**
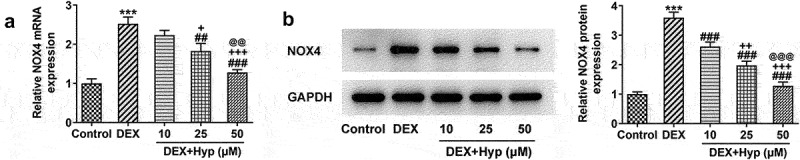
Hyp down-regulates NOX4 expression in DEX-induced MC3T3-E1 osteoblasts.NOX4 expression at mRNA level (A) and protein level (B) in MC3T3-E1 osteoblasts was measured by RT-qPCR and western blot, respectively. ***P < 0.001 vs control; ^##^P < 0.01 and ^###^P < 0.001 vs DEX. ^+^P < 0.05, ^++^P < 0.01 and ^+++^P < 0.001 vs DEX+Hyp (10 μM). ^@@^P < 0.01 and ^@@@^P < 0.001 vs DEX+Hyp (25 μM).

Thereafter, we investigated whether Hyp could regulate NOX4 expression in osteoblasts. As shown in Figure 3, NOX4 expression at both mRNA and protein levels was significantly up-regulated by DEX. Nevertheless, the upregulated expression of NOX4 caused by DEX was markedly reduced upon Hyp co-treatment, suggesting that Hyp could inhibit NOX4 expression in DEX-treated osteoblasts.

**Figure 4.**
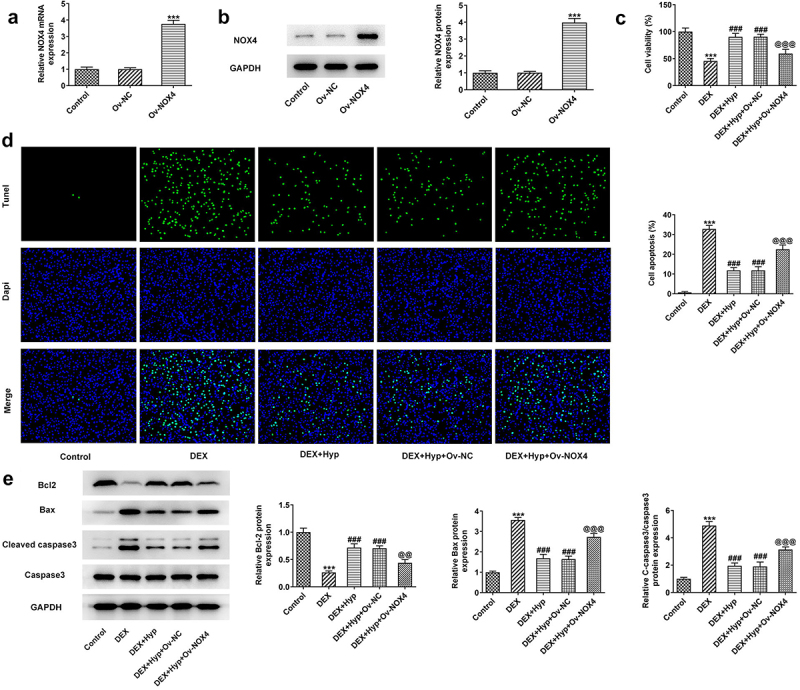
NOX4 overexpression reverses the effect of Hyp on MC3T3-E1 osteoblasts survival and apoptosis.A-B, NOX4 was overexpressed in MC3T3-E1 osteoblasts via transfection of Ov-NOX4, and then RT-qPCR and western blot were utilized to test NOX4 expression. C, cell viability was appraised by CCK-8. D, the apoptotic capacity was observed by Tunel (x200). E, protein levels of Bcl2, Bax and cleaved caspase 3/caspase 3 were detected by western blot assay. ***P < 0.001 vs control; ^###^P < 0.001 vs DEX; ^@@^P < 0.01 and ^@@@^P < 0.001 vs DEX + Hyp + Ov-NC.

**Figure 5.**
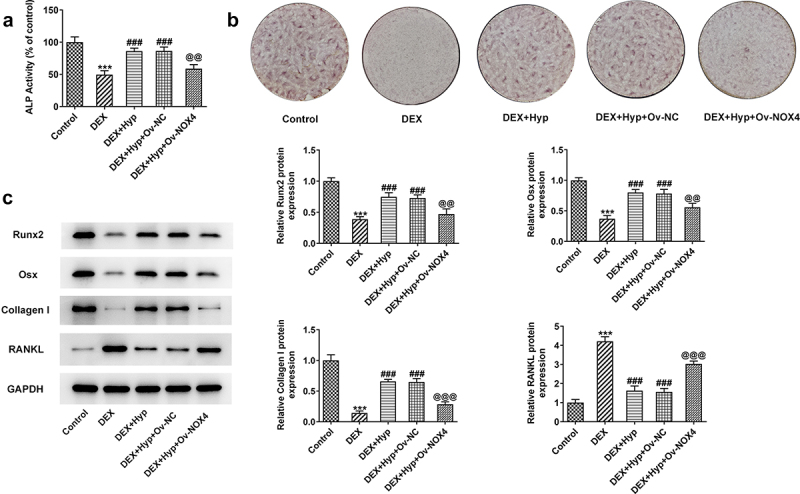
NOX4 overexpression reverses the impacts of Hyp on MC3T3-E1 osteoblasts differentiation.A, ALP assay measured ALP activity. B, osteogenic differentiation was examined by ARS staining. C protein levels of Runx2, Osx, Collagen I and RANKL were analyzed by western blot assay. ***P < 0.001 vs control; ^###^P < 0.001 vs DEX; ^@@^P < 0.01 and ^@@@^P < 0.001 vs DEX + Hyp + Ov-NC.

To verify whether Hyp exerted its protective effect against DEX-induced osteoblasts injury via inhibiting NOX4 expression, we overexpressed NOX4 in MC3T3-E1 osteoblasts via transfection with NPX4 overexpression vector (Ov-NOX4). Results in Figure 4(a,b) validated the significant up-regulation of NOX4 at both mRNA and protein levels in cells. Subsequently, MC3T3-E1 osteoblasts that were transfected with NOX4 overexpression plasmids or not were co-treated with DEX and 50 μM Hyp. As shown in Figure 4(c), cells transfected with Ov-NOX4 exhibited significantly reduced cell viability in the presence of DEX and Hyp co-treatment compared with that in cells transfected with Ov-NC. In addition, the apoptosis inhibited by Hyp in DEX-insulted MC3T3-E1 osteoblasts was markedly exacerbated by NOX4 overexpression (Figure 4(d)). Consistently, the influence of Hyp on Bcl2, Bax, and cleaved caspase3/caspase3 protein levels in DEX-treated cells was also blocked by NOX4 overexpression (Figure 4(e)). Moreover, in comparison with DEX + Hyp + Ov-NC group, cells in DEX + Hyp + Ov-NOX4 group showed marked decrease in ALP activity and the number of calcium nodule, indicating the inhibition of osteogenic differentiation imposed by NOX4 elevation (Figure 5(a-b)). Similar results are observed in Figure 5(c), which showed that NOX4 overexpression reversed the effects of Hyp on Runx2, Osx, Collagen I, and RANKL protein expressions.

Hyp inhibited the activation of ROS/JNK signaling in DEX-treated MC3T3-E1 osteoblasts via reducing NOX4 expression.

**Figure 6.**
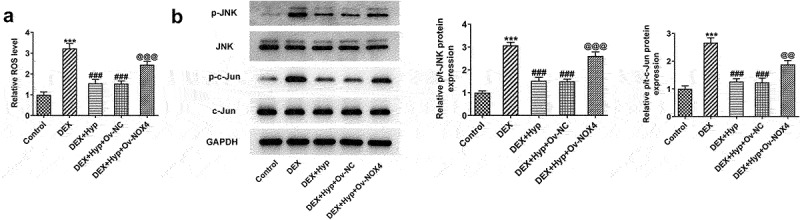
Hyp inhibits the activation of ROS/JNK signaling in DEX-treated MC3T3-E1 osteoblasts via reducing NOX4 expression.A, ROS generation was detected by ROS Assay. B, p p-JNK/JNK and p-c-Jun/c-Jun protein levels were analyzed by western blot assay. ***P < 0.001 vs control; ^###^P < 0.001 vs DEX; ^@@^P < 0.01 and ^@@@^P < 0.001 vs DEX + Hyp + Ov-NC.

Finally, we aimed to uncover the involved signaling underlying the actions of Hyp. We found that DEX led to the increase in ROS level and p-JNK/JNK and p-c-Jun/c-Jun protein levels, suggesting the stimulation of ROS/JNK signaling by DEX (Figure 6(a,b)). However, Hyp co-treatment blocked the effects caused by DEX. In addition, after NOX4 was overexpressed, ROS level and p-JNK/JNK and p-c-Jun/c-Jun expression in DEX + Hyp-treated cells were significantly enhanced (Figure 6(a,b)). These results indicated that the inhibitory effects of Hyp on the activation of ROS/JNK pathway caused by DEX in MC3T3-E1 osteoblasts depended on the down-regulating of NOX4 expression.

## Discussion

Hyp belongs to flavonoids and has wide existence in Chinese herbal medicines [16]. The inhibitory effects of Hyp on inflammation, infection, oxidative stress, and tumor growth have been extensively studied since 1950s [7,17]. In the present study, we demonstrated that Hyp could effectively protect osteoblasts from DEX-induced impairment of proliferation and differentiation. Further experiments showed that the effects of Hyp on osteoblasts depended on the down-regulating of NOX4 expression, thereby inactivating ROS/JNK signaling pathway.

Emerging evidences have shown that herbal-based phytotherapy exhibited effective effects on the treatment of bone injuries and bone-related diseases [18,19]. For instance, extracts from safflower seeds were confirmed to accelerate osteoblast differentiation in MC3T3-E1 cells [20]. Deoxyactein, an extract from Chinese herbal black cohosh, was demonstrated to stimulate osteoblast function and inhibit bone-resorbing mediators in MC3T3-E1 cells [21]. The bioactive compound of Cordyceps militaris, cordycepin, could inhibit RANKL-induced osteoclast differentiation [22]. It is known that in bone microenvironments, the impaired bone cell survival, bone formation, as well as the promoted osteoclastic resorption and the damaged osteoclasts differentiation could result in the occurrence of ONFH [23]. Therefore, the above reports suggested the potential value of Chinese medicine in the treatment of ONFH. Herein, we showed that in DEX-induced GONFH cell model in vitro, Hyp promoted survival and inhibited apoptosis of MC3T3-E1 osteoblast in a concentration-dependent manner. The osteoblast differentiation ability that damaged by DEX was also improved by Hyp, as reflected by elevated ALP activity, increased number of calcium nodule, enhanced expressions of Runx2, Osx, and collagen I, as well as reduced expression of RANKL. It is widely accepted that osteoblast differentiation is characterized by the synthesis of osteopontin, Runx2, Osx, and collagen I, while RANKL signaling is responsible for osteoclasts formation and survival [24]. Therefore, our results revealed that Hyp may attenuate GONFH via improving the survival and differentiation of osteoblasts to promote bone formation.

To figure out the mechanisms behind the actions of Hyp on GONFH, we identified NOX4 as the downstream target of Hyp after searching SwissTargetPrediction online database. NOX4 belongs to the NOX family, which is expressed on the cytoplasm membrane and is known to produce ROS through passing electrons [25]. Enhanced ROS level has been shown to play a role in ONFH [26]. Our results manifested that Hyp markedly inhibited NOX4 expression in DEX-induced osteoblasts, confirming the regulation of Hyp on NOX4 expression. To verify whether Hyp exerted its effect on GONFH via down-regulating NOX4 expression, we overexpressed NOX4 in Hyp and DEX co-treated osteoblasts. In compliance with our hypothesis, the protective role of Hyp in DEX-induced osteoblasts was significantly reversed by overexpression of NOX4. Moreover, Hyp also impeded ROS generation, but this effect was then blocked by NOX4 overexpression. Hyp has been documented to exert its anti-oxidative effects through inhibiting ROS production [27]. Besides, ROS is able to stimulate JNK/c-Jun pathway [28]. A recent study reported that glucocorticoids induced ONFH in rats via ROS/JNK/c-Jun pathway [14]. We subsequently observed that Hyp inhibited the activation of JNK pathway caused by DEX, and NOX4 blunted this effect. These observations illustrated that Hyp could inactivate ROS/JNK signaling via suppressing NOX4 expression in DEX-induced osteoblasts.

## Conclusion

Taken together, our results illustrated that Hyp could improve the survival and osteogenic differentiation of DEX-induced osteoblasts via down-regulating NOX4 expression to inhibit ROS/JNK signaling. This study for the first time evidenced that Hyp may be a potent candidate drug for GONFH therapy. Considering the environments in vivo and in vitro are different, additional animal experiments with glucocorticoid-induced osteoporosis need to be explored in future studies.

## Data Availability

The datasets used and/or analysed during the current study are available from the corresponding author on reasonable request.

## List of abbreviations

Osteonecrosis of the femoral head (ONFH);

Glucocorticoid-induced ONFH (GONFH);

Hyperoside (Hyp);

NADPH Oxidase 4 (NOX4);

reactive oxygen species (ROS);

c-Jun N-terminal kinase (JNK);

dexamethasone (DEX);

Runt-related transcription factor 2 (Runx2);

receptor activator nuclear factor κB ligand (RANKL);

Alkaline phosphatase (ALP).

## References

[1] Wu X, Sun W, Tan M. Noncoding RNAs in Steroid-Induced Osteonecrosis of the Femoral Head. Biomed Res Int. 2019;2019:8140595. 31930139 10.1155/2019/8140595 PMC6942769

[2] Wang A, Ren M, Wang J. The pathogenesis of steroid-induced osteonecrosis of the femoral head: a systematic review of the literature. Gene. 2018;671:103–109. 29859289 10.1016/j.gene.2018.05.091

[3] Chang C, Greenspan A, Gershwin ME. The pathogenesis, diagnosis and clinical manifestations of steroid-induced osteonecrosis. J Autoimmun. 2020;110:102460. 32307211 10.1016/j.jaut.2020.102460

[4] Zhan J, Yan Z, Zhao M, et al. Allicin inhibits osteoblast apoptosis and steroid-induced necrosis of femoral head progression by activating the PI3K/AKT pathway. Food Funct. 2020;11(9):7830–7841. 32808945 10.1039/d0fo00837k

[5] Wei A, Song Y, Ni T, et al. Hyperoside attenuates pregnancy loss through activating autophagy and suppressing inflammation in a rat model. Life Sci. 2020;254:117735. 32360572 10.1016/j.lfs.2020.117735

[6] Qiu J, Zhang T, Zhu X, et al. Hyperoside Induces Breast Cancer Cells Apoptosis via ROS-Mediated NF-κB Signaling Pathway. Int J Mol Sci. 2019;21(1):131.

[7] Huang J, Tong X, Zhang L, et al. Hyperoside Attenuates Bleomycin-Induced Pulmonary Fibrosis Development in Mice. Front Pharmacol. 2020;11:550955. 33192501 10.3389/fphar.2020.550955 PMC7642689

[8] Zhang N, Ying MD, Wu YP, et al. Hyperoside, a flavonoid compound, inhibits proliferation and stimulates osteogenic differentiation of human osteosarcoma cells. PloS one. 2014;9(7):e98973. 24983940 10.1371/journal.pone.0098973 PMC4077650

[9] Chen Y, Dai F, He Y, et al. Beneficial effects of hyperoside on bone metabolism in ovariectomized mice. Biomed Pharmacothe. 2018;107:1175–1182.

[10] Xu T, Wu X, Zhou Z, et al. Hyperoside ameliorates periodontitis in rats by promoting osteogenic differentiation of BMSCs via activation of the NF-κB pathway. FEBS Open Bio. 2020;10(9):1843–1855.

[11] Yan Z, Zhan J, Qi W, et al. The Protective Effect of Luteolin in Glucocorticoid-Induced Osteonecrosis of the Femoral Head. Front Pharmacol. 2020;11:1195. 32903480 10.3389/fphar.2020.01195 PMC7435053

[12] Kuang MJ, Zhang WH, He WW, et al. Naringin regulates bone metabolism in glucocorticoid-induced osteonecrosis of the femoral head via the Akt/Bad signal cascades. Chem Biol Interact. 2019;304:97–105. 30878453 10.1016/j.cbi.2019.03.008

[13] Wang XY, Gong LJ, Huang JM, et al. Pinocembrin alleviates glucocorticoid-induced apoptosis by activating autophagy via suppressing the PI3K/Akt/mTOR pathway in osteocytes. Eur J Pharmacol. 2020;880:173212. 32470335 10.1016/j.ejphar.2020.173212

[14] Peng P, Nie Z, Sun F, et al. Glucocorticoids induce femoral head necrosis in rats through the ROS/JNK/c-Jun pathway. FEBS Open Bio. 2021;11(1):312–321.

[15] Fan ZQ, Bai SC, Xu Q, et al. Oxidative Stress Induced Osteocyte Apoptosis in Steroid-Induced Femoral Head Necrosis. Orthop Surg. 2021;13(7):2145–2152. 34559465 10.1111/os.13127 PMC8528976

[16] Farnsworth NR, Wagner H, Hörhammer L, et al. Aster pilosus (Compositae). I. Isolation of hyperoside (quercetin-3-beta-D-mono-galactoside) from the leaves. J Pharm Sci. 1968;57(6):1059–1061. 5677684 10.1002/jps.2600570641

[17] Li S, Zhang Z, Cain A, et al. Antifungal activity of camptothecin, trifolin, and hyperoside isolated from Camptotheca acuminata. J Agric Food Chem. 2005;53(1):32–37. 15631505 10.1021/jf0484780

[18] Wong RW, Rabie AB. Traditional Chinese medicines and bone formation–a review. J Oral Maxillofacial Surg. 2006;64(5):828–837.

[19] Che CT, Wong MS, Lam CW. Natural Products from Chinese Medicines with Potential Benefits to Bone Health. Molecules (Basel Switzerland). 2016;21(3):239.

[20] Jang HO, Park YS, Lee JH, et al. Effect of extracts from safflower seeds on osteoblast differentiation and intracellular calcium ion concentration in MC3T3-E1 cells. Nat Prod Res. 2007;21(9):787–797. 17654282 10.1080/14786410601133475

[21] Choi EM. Deoxyactein stimulates osteoblast function and inhibits bone-resorbing mediators in MC3T3-E1 cells. J Appl Toxicol. 2013;33(3):190–195. 21910134 10.1002/jat.1733

[22] Kim J, Lee H, Kang KS, et al. Cordyceps militaris mushroom and cordycepin inhibit RANKL-induced osteoclast differentiation. J Med Food. 2015;18(4):446–452. 25789604 10.1089/jmf.2014.3215

[23] Zalavras CG, Lieberman JR. Osteonecrosis of the femoral head: evaluation and treatment. J Am Acad Orthop Surg. 2014;22(7):455–464. 24966252 10.5435/JAAOS-22-07-455

[24] Boyce BF, Xing L. Functions of RANKL/RANK/OPG in bone modeling and remodeling. Arch Biochem Biophys. 2008;473(2):139–146. 18395508 10.1016/j.abb.2008.03.018 PMC2413418

[25] Huang C, Gan D, Luo F, et al. Interaction Mechanisms Between the NOX4/ROS and RhoA/ROCK1 Signaling Pathways as New Anti- fibrosis Targets of Ursolic Acid in Hepatic Stellate Cells. Front Pharmacol. 2019;10:431. 31130857 10.3389/fphar.2019.00431 PMC6510285

[26] Deng S, Zhou JL, Fang HS, et al. Sesamin Protects the Femoral Head From Osteonecrosis by Inhibiting ROS-Induced Osteoblast Apoptosis in Rat Model. Front Physiol. 2018;9:1787. 30618801 10.3389/fphys.2018.01787 PMC6298420

[27] Liu Z, Tao X, Zhang C, et al. Protective effects of hyperoside (quercetin-3-o-galactoside) to PC12 cells against cytotoxicity induced by hydrogen peroxide and tert-butyl hydroperoxide. Biomed Pharmacothe. 2005;59(9):481–490.

[28] Suzuki M, Bandoski C, Bartlett JD. Fluoride induces oxidative damage and SIRT1/autophagy through ROS-mediated JNK signaling. Free Radic Biol Med. 2015;89:369–378. 26431905 10.1016/j.freeradbiomed.2015.08.015 PMC4684823

